# Protease-Resistant, Broad-Spectrum Antimicrobial Peptides with High Antibacterial and Antifungal Activity

**DOI:** 10.3390/life15020242

**Published:** 2025-02-05

**Authors:** Tanil Kocagoz, Betul Zehra Temur, Nihan Unubol, Merve Acikel Elmas, Zeynep Kanlidere, Sumeyye Cilingir, Dilan Acar, Gizem Boskan, Sumeyye Akcelik Deveci, Esma Aybakan, Aslihan Ozcan Yoner, Neval Yurttutan Uyar, Mustafa Serteser, Seray Sahsuvar, Yigit Erdemgil, Zeynep Zulfiye Yildirim Keles, Deniz Demirhan, Sandra Sakalauskaite, Rimantas Daugelavicius, Tugba Arzu Ozal Ildeniz, Ahmet Emin Atik, Erkan Mozioglu, Tarik Eren, Serap Arbak, Guldal Suyen, Ozge Can

**Affiliations:** 1 Department of Medical Biotechnology, Institute of Health Sciences, Acibadem Mehmet Ali Aydinlar University, Istanbul 34752, Turkey; betulzkarakus@gmail.com (B.Z.T.); smyy.akclk@gmail.com (S.A.D.); esmaaybakan@hotmail.com (E.A.); seray.sah@gmail.com (S.S.); erkan.mozioglu@acibadem.edu.tr (E.M.); 2 Department of Medical Microbiology, School of Medicine, Acibadem Mehmet Ali Aydinlar University, Istanbul 34752, Turkey; neval.uyar@acibademlabmed.com.tr; 3 Medical Laboratory Techniques, Vocational School of Health Services, Acibadem Mehmet Ali Aydinlar University, Istanbul 34752, Turkey; nihan.unubol@acibadem.edu.tr; 4 Department of Histology and Embryology, School of Medicine, Acibadem Mehmet Ali Aydinlar University, Istanbul 34752, Turkey; merve.elmas@acibadem.edu.tr (M.A.E.); serap.arbak@acibadem.edu.tr (S.A.); 5 Department of Basic Pharmaceutical Sciences, Faculty of Pharmacy, Acibadem Mehmet Ali Aydinlar University, Istanbul 34752, Turkey; zeynep.kanlidere@acibadem.edu.tr; 6 Department of Physiology, Institute of Health Sciences, Acibadem Mehmet Ali Aydinlar University, Istanbul 34752, Turkey; sumeyye.clngr@gmail.com (S.C.); dilan.acar@live.acibadem.edu.tr (D.A.); 7 Department of Biomedical Engineering, Institute of Natural Sciences, Acibadem Mehmet Ali Aydinlar University, Istanbul 34752, Turkey; gzmboskan@gmail.com (G.B.); aslihanozcan93@gmail.com (A.O.Y.); 8 Department of Medical Biochemistry, School of Medicine, Acibadem Mehmet Ali Aydinlar University, Istanbul 34752, Turkey; mustafa.serteser@acibademlabmed.com.tr; 9 Turgut Ilaclari A.S., Kocaeli 41400, Turkey; yerdemgil@turgutilac.com.tr (Y.E.); zkeles@turgutilac.com.tr (Z.Z.Y.K.); denizbaycin@gmail.com (D.D.); aatik@turgutilac.com.tr (A.E.A.); 10 Department of Biochemistry, Faculty of Natural Sciences, Vytautas Magnus University, 44248 Kaunas, Lithuania; sandra.sakalauskaite@lsmu.lt (S.S.); rimantas.daugelavicius@vdu.lt (R.D.); 11 Laboratory of Immunology, Department of Immunology and Allergology, Lithuanian University of Health Sciences, 44307 Kaunas, Lithuania; 12 Department of Biomedical Engineering, Faculty of Engineering and Natural Sciences, Acibadem Mehmet Ali Aydinlar University, Istanbul 34752, Turkey; tugba.ildeniz@istinye.edu.tr; 13 Department of Natural Sciences, Faculty of Engineering and Natural Sciences, Acibadem Mehmet Ali Aydinlar University, Istanbul 34752, Turkey; 14 Department of Chemistry, Faculty of Engineering and Natural Sciences, Yildiz Technical University, Istanbul 34220, Turkey; erentari@gmail.com; 15 Department of Physiology, School of Medicine, Acibadem Mehmet Ali Aydinlar University, Istanbul 34752, Turkey; guldal.suyen@acibadem.edu.tr

**Keywords:** protein-mimicking peptides, peptide antibiotics, peptide synthesis, antimicrobial peptides, protease resistance, multidrug resistance

## Abstract

Antimicrobial peptides (AMPs) are a diverse group of small, naturally occurring molecules that orchestrate the innate immune response of various organisms, from microorganisms to humans. Characterized by their broad-spectrum activity against bacteria, fungi and viruses, AMPs are increasingly recognized for their potential as novel therapeutic agents in the face of rising antibiotic resistance. Here, we present several newly designed AMPs, one of which, DTN6, exerts significant activity against several organisms with MIC values as low as 0.5 µg/mL. The D-TN6 peptide influences both bacteria and yeasts. Scanning electron microscopy and transmission electron microscopy results showed that the bacterial membrane is affected by D-TN6, which is resistant to proteases and is effective against antibiotic-resistant pathogens with hemolytic activity and low toxicity. The D-TN6 peptide is effective in vivo against standard *S. aureus* strains in wounds. Thus, D-TN6 is a potent antibiotic candidate with a broad spectrum of activity. Overall, AMPs are a promising tool for the development of next-generation antimicrobial agents that could mitigate global health threats posed by multidrug-resistant pathogens.

## 1. Introduction

In the last few decades, an increasing number of bacterial species have developed resistance to the antibiotics currently in use. Nearly 5 million deaths associated with antimicrobial resistance were recorded worldwide in 2019, and 10 million could die annually in 2050 if a radical solution remains to be found [1]. In addition, fungal infections cause 1.5 million deaths annually, and antifungal drug resistance increases every year [2]. Consequently, research has increasingly focused on antimicrobial peptides, against which bacteria are less likely to develop resistance [2,3,4]. A growing number of clinical trials for a large number of AMPs have taken place to see if they can replace conventional antibiotics [2].

Antimicrobial peptides (AMPs) are bioactive amino acid sequences that comprise the natural defense system of living organisms. Recently, AMPs have attracted attention as innovative drug candidates and novel immunomodulatory therapies for the treatment of infectious diseases [5]. They function by disrupting bacterial cell membranes, modulating immune responses, and regulating inflammation [6]. AMPs are divided into two main groups: defensins and cathelicidins. Approximately 30 different cathelicidin derivatives have been identified in mammalian species. In humans, a single cathelicidin species, known as the 18-kDa human cationic antibacterial protein (hCAP18), has been found. hCAP18 includes an LL-37 region and an antibacterial peptide composed of 37 amino acid residues starting with two leucine residues (LLGDFFRKSKEKIGKEFKRIVQRIKDFLRNLVPRTES), and it is primarily found in epithelial cells and neutrophils [7].

The primary mechanisms of action of AMPs include membrane permeabilization and structural disruption. AMPs exhibit amphipathic properties in nonpolar environments; one of their most significant characteristics is their recurring positive charges, which enable them to approach and interact with the negatively charged bacterial membrane [8]. Bacterial membranes tend to be negatively charged due to the anionic nature of the main components of bacterial membranes such as CL (cardiolipin), PG (phosphatidylglycerol), LPS (lipopolysaccharide), teichoic (TA) and lipoteichoic acids (LTA), and phospholipids that possess negatively charged head groups [9]. Together with these, anionic lipids allow cationic AMPs to selectively target bacteria against host cell membranes [10]. The outer surface of fungi is also negatively charged. In addition, these peptides tend to target fungi over mammalian cells due to the presence of sterols (e.g., ergosterol) in fungal membranes [11]. What drives the movement of these peptides toward pathogens is the electrostatic attraction arising from the opposite charges between AMPs and membranes. The effect on the membrane makes it difficult for microorganisms with compromised cellular integrity to develop resistance [10,12].

The most significant disadvantage of AMPs is their sensitivity to proteases [11]. However, AMPs can be made protease-resistant by introducing D-form amino acids into the AMP sequence. It has been shown that AMPs synthesized in both the L- and D-forms exhibit similar antimicrobial activity [13,14]. Based on our prior experience, peptides synthesized in the D-form demonstrated even better activity than those synthesized in the L-form.

There are 27 small peptides that are less than 50 amino acids long and that are in the FDA drug database; 7 of these small peptides are AMPs [15]. Vancomycin, one of these small peptides, is a branched tricyclic glycosylated peptide antibiotic that is effective against Gram-positive pathogenic bacteria. In the clinic, it represents the last line of defense because it elicits toxic side effects [16].

In this study, we designed AMPs by utilizing the conserved sequences of cathelicidin and rendering them protease-resistant. These peptides showed strong antibacterial and antifungal activity; it was not possible to obtain laboratory mutants resistant to these peptides. We also showed that these peptides were effective in treating bacterial skin infections and wound healing in a rat model. The peptides developed in this study are therefore therapeutic candidates for the treatment of infections, especially those that are multidrug-resistant.

## 2. Materials and Methods

### 2.1. Materials

#### 2.1.1. Peptide Synthesis and Analysis

All chemicals and reagents were of analytical grade unless otherwise stated. HPLC-grade acetonitrile was purchased from Merck (Darmstadt, Germany). Anhydrous dimethylformamide (DMF), diisopropylcarbodiimide (DIC), Oxyma^®^, piperidine, dichloromethane (DCM), and trifluoroacetic acid (TFA) were obtained from Sigma-Aldrich (Darmstadt, Germany).

#### 2.1.2. In Vitro Experiments

Mueller–Hinton agar (MHA) (Oxoid, Hampshire, UK), Mueller–Hinton broth (MHB) (Oxoid, Hampshire, UK), ampicillin (GenemarkBio, Taichung City, Taiwan), Dulbecco’s modified Eagle’s medium (DMEM) (Gibco™, Langenselbold, Germany), fetal bovine serum (FBS) (ATCC 30-2020™, Manassas, VA, USA), penicillin–streptomycin (Pen/Strep) (Gibco™, Langenselbold, Germany), magainin-2 (Mag-2) (Sigma-Aldrich, St. Louis, MO, USA), Triton X-100 (Sigma-Aldrich, St. Louis, MO, USA), proteinase k (Qiagen, Hilden, Germany), and the Pierce™ Quantitative Fluorometric Peptide Assay (Thermo Scientific™, San Jose, CA, USA) were used.

#### 2.1.3. Bacterial Strains and Cells

*Staphylococcus aureus* (*S. aureus*, ATCC 29213) as a Gram-positive coccus, *Escherichia coli* (*E. coli*, ATCC 25922) as a fermentative Gram-negative bacillus, *Pseudomonas aeruginosa (P. aeruginosa*, ATCC 27853) as a non-fermentative Gram-negative bacillus, *Candida albicans (C. albicans*, ATCC 10231) as a yeast fungi, and *E. coli* DH5α, KMY1 (MK12 λRS45 [Φ(emrK‘-lacZ)]), and KMA1 (KMY1 acrA::cat) were used as bacterial strains. Clinically resistant bacterial strains that are classified as “ESKAPE pathogens”, including vancomycin-resistant *Enterococcus faecium* (VRE), methicillin-resistant *S. aureus* (MRSA), *Klebsiella pneumoniae* (KP)*, Acinetobacter baumannii* (AB), *P. aeruginosa* (PA) by WHO21, multi-drug-resistant *Aspergillus flavus* (AF), *C. albicans* (CA), and *C. krusei* (CK), were provided by Acibadem Labmed.

Mouse embryonic fibroblasts (3T3, CRL-1658™, ATCC^®^) and human skin keratinocytes (HaCaT, PCS-200-011™, ATCC^®^) were also used for cell culture experiments.

#### 2.1.4. Animals

Wistar rats (250–300 g; 12 weeks; male) were obtained from the Acibadem Mehmet Ali Aydinlar University Laboratory Animal Research Center (ACUDEHAM). Rats were kept under controlled conditions (12 h/12 h light/dark cycles, temperature of 22 ± 2 °C, humidity of 65–70%) and fed with standard pellet and tap water ad libitum. All protocols for animal experiments were approved by the Acibadem Mehmet Ali Aydinlar University Local Ethics Committee (No. HDK-2017/16, 23 April 2017). All treatments were performed in accordance with the ARRIVE guidelines and the Guide to the Care and Use of Laboratory Animals, Eighth Edition [17,18].

#### 2.1.5. In Vivo Experiments

A Relassay Myeloperoxidase Peroxidation Activity Assay Kit and total antioxidant status and total oxidant status measurement kits were purchased from Rel Assay, Turkey. TNF-α, IL1-β, and IL-6 level measurement kits were purchased from Sunred, China. Superoxide dismutase level and glutathione level measurement kits were purchased from Elabscience, Houston, TX, USA.

### 2.2. Methods

#### 2.2.1. Design of the Synthesized Peptides

AMPs were designed by mimicking the C-terminal of the cathelicidin LL-37 molecule, which is known to have antimicrobial activity. AMPs have an alpha helix structure with hydrophobic leucine amino acids and positively charged arginine amino acids that target the bacterial cell membrane. Peptides with Val, Ile, or Ala amino acids, which are also hydrophobic and replace Leu amino acids, were designed and synthesized (Table 1).

#### 2.2.2. Molecular Dynamics Simulations

##### Modeling the L-Form Peptides

The 3D structures of the designed L-form peptides were obtained using the PEP-FOLD3 server [19], and MD simulations with CHARMM force field parameters were performed by running the NAMD 2.11 software in parallel [20]. Peptide molecules were placed above a model POPE membrane at a certain distance via VMD (version 1.9) software scripting [21]. After the lipid membrane system and peptides reached equilibrium, the entire system was ready for MD simulations, which were performed for 100 ns. The outputs were visualized with the VMD software and analyzed via scripts.

##### Modeling of the D-Form Peptides

As it was not possible to obtain the 3D structure of the designed D-form proteins with the PEP-FOLD3 server and similar tools, a code was developed based on the coordinates of the alpha helix structure of the L-form to obtain the 3D structure of the D-form of the TN1 peptide molecule. A new topology file for the D-form [22] was prepared, and the NAMD 2.11 software [20] was run in parallel so that the MD simulations were carried out for the peptides designed in the D-form, with the 300 ns long MD simulation extended by 300 ns for a total of 600 ns.

#### 2.2.3. Peptide Synthesis and Characterization

All peptides in Table 1 were synthesized following the method of Bulut et. al. using a peptide synthesizer (CEM, Liberty™ Blue and CEM Discover™, Matthews, NC, USA) [23]. The primary peptide sequences were verified using a high-resolution mass spectrometer. The system consisted of a UPLC (Acquity H-Class Bio) coupled to a Xevo^®^ G2-XS QToF mass spectrometer (Waters, Milford, MA, USA) with an ESI source, operated in the positive ion mode. System control, data acquisition, and data processing were conducted using the UNIFI™ software (version 1.8.2). Peptides were identified by comparing the accurate molecular mass and MS/MS fragment ions with the expected amino acid sequence of the peptide.

#### 2.2.4. Minimum Inhibitory Concentration

The antibacterial activity of the bacterial strains was determined using the EUCAST broth microdilution method [24]. Bacterial strains were streaked into the MHA and incubated at 37 °C overnight. The initial doses were adjusted to 1024 µg/mL using a peptide concentration measurement kit (Pierce™ Quantitative Fluorometric Peptide Assay, Thermo Scientific™) and serially diluted to 0.125 µg/mL. All samples were tested in triplicate [25,26]. The antifungal activity of the Candida species was measured in accordance with the guidelines in CLSI document M27-A3 [27].

#### 2.2.5. Cell Cytotoxicity

3T3 and HaCaT cells were grown in DMEM consisting of 1% pen/strep and 10% FBS. A total of 5 × 10^4^ cells were loaded into each well of a 96-well plate and incubated at 37 °C using a 5% CO_2_ incubator. All samples were measured in triplicate between 0.5–32 µg/mL concentrations and incubated at 37 °C for 24 h in a 5% CO_2_ incubator. Only cells and Mag-2 were used as negative and positive controls, respectively. After 24 h, cell cytotoxicity was evaluated using a cell proliferation kit (MTT, Roche, Basel, Switzerland) following the kit protocol and analyzed at 550 nm and 690 nm using a microplate reader (Gen5 Synergy HT BioTek) [3]. The concentration of peptides that killed 50% of the cells was determined as the IC50 in the cytotoxicity experiments.

#### 2.2.6. Hemolytic Activity

Permission to acquire the fresh human blood used for this experiment was granted by the Acibadem Mehmet Ali Aydinlar University Local Ethics Committee (No. 2023-2/32, 27 January 2023). First, 30 µL of fresh male human blood was suspended with 10 mL of autoclaved Tris saline buffer (TBS buffer, 10 mM Tris, 150 mM NaCl, pH 7.2) and centrifuged thrice for 5 min at 1500 rpm. Peptide concentrations between 0.5–32 µg/mL (100 µL total) were serially diluted, added to 100 µL of blood solution, and incubated at 37 °C for 30 min. Three replicates were performed for each peptide dose. A sample of 20% Triton-X 100/DMSO was used as a positive control. After incubation, the 96-well plate was centrifuged at 1500 rpm for 10 min. Absorbance was measured at 414 nm using a microplate reader (Gen5 Synergy HT BioTek) [25]. The % lysis of the peptide was calculated as follows:

% lysis = OD_414_ − OD_414_ (blank)/OD_414_ (total lysis − blank) × 100

#### 2.2.7. Stability Toward Enzymatic Degradation

Peptide solutions were prepared at a final concentration of 1 mg/mL in TBS buffer (50 mM Tris, 150 mM NaCl, pH 7.6) including 10% DMSO. The proteinase K solution was adjusted to 1 mg/mL (enzyme activity = 30 U/mg). The peptide solution was then incubated at 37 °C overnight in an orbital shaker. After incubation, the reaction was blocked by adding 1% TFA (100 µL) in water [28]. Peptide solutions with/without the proteinase K enzyme were analyzed at 214 nm using HPLC with an analytic column (AdvanceBio Peptide Plus, 2.1 × 150 mm, 2.7 µm, LC column, Agilent Technologies, Santa Clara, CA, USA) following the linear gradient method (30 min in a range of 5–80% acetonitrile including 0.025% TFA). MIC experiments were repeated to measure biological activities after treating the peptides with proteinase K.

#### 2.2.8. Resistance Development Studies

The MIC assay was established using *S. aureus* ATCC 29213. The resistance development rates of the bacteria using D-TN6 and gentamicin, which are required to inhibit *S. aureus*, were analyzed by measuring the MIC values after serial passages. The experiment was performed between concentrations of 0.125 µg/mL and 128 µg/mL. The 96-well plate in which the MIC was established was incubated at 37 °C for 4 h. Then, each well was plated on MHA plates and left overnight at 37 °C. The MIC assay was repeated with bacteria grown at the highest D-TN6 and gentamicin concentrations until resistance developed.

#### 2.2.9. Electrochemical Measurements

Potentiometric measurements of the TPP+ concentration in the incubation medium were performed simultaneously in two thermostated reaction vessels using *E. coli* DH5*α*; KMY1 (MK12 λRS45 [Φ (emrK ‘-lacZ)]) and KMA1 (KMY1 acrA: cat); and *S. aureus* ATCC 29,213 bacteria. The experiments were performed at 37 °C in 5 mL of magnetically stirred medium. The concentrated cell suspension was added to obtain an OD600 of 1. TPP+-selective electrodes were connected to the potential-amplifying system based on an ultralow input bias current operational amplifier AD549JH (Analog Devices, Norwood, MA, USA). The amplifying system was connected to a computer through a PowerLab 4/35 logger (AD Instruments Pty Ltd., Bella Vista, Australia). Ag/AgCl reference electrodes (model Orion 9001, Thermo Inc., Cambridge, MA, USA) were indirectly connected to the measuring vessels by agar salt bridges. Polymyxin B, which disrupts the outer cell membrane of Gram-negative bacteria, was used as a control. A curve with increasing concentrations of polymyxin B under the same conditions was used for comparison. Cells were incubated in 100 mM Tris/HCl buffer, pH 8.0, 0.1% glucose, supplemented with or without MgCl_2_.

#### 2.2.10. Scanning Electron Microscopy (SEM)

For the SEM analysis, a dialysis membrane was used to capture *E. coli* ATCC 25922. Bacterial samples were fixed overnight in 2.5% glutaraldehyde fixative with 0.1M phosphate-buffered saline (PBS, pH 7.2), and post-fixation was performed with 1% osmium tetroxide for 1 h. *E. coli* samples were dehydrated with increasing concentrations (70%, 90%, 96%, 100%) of ethyl alcohol. After dehydration, the samples were placed in an alcohol/amyl acetate series and then dried in a critical point dryer and sputtered with gold [29]. *E. coli* samples were then examined under SEM (Thermo Scientific—Quattro SEM) at magnifications of (A) 10,000X, (B) 25,000X, (C) 100,000X, (D) 120,000X, (E) 150,000X, and (F) 200,000X.

#### 2.2.11. Transmission Electron Microscopy (TEM)

*E. coli* ATCC 25,922 samples were fixed in 2.5% glutaraldehyde fixative with 0.1 M PBS buffer (pH 7.2). Then, the bacterial samples were centrifuged at 300× *g* for 5 min. The cell pellet was rinsed with 0.1 M PBS before embedding in agar. Next, the samples were post-fixed with 1% osmium tetroxide for 1 h. Bacterial samples were dehydrated with increasing concentrations of ethyl alcohol and embedded in Epon 812 resin (Electron Microscopy Science, Hatfield, PA, USA) [30]. Ultrathin sections were negatively stained with 2% uranyl acetate solution and then examined with TEM (Thermo Scientific—Talos L120C).

#### 2.2.12. Animal Experiment Design

Two different wound models were used to determine the therapeutic effect of the D-TN6 peptide. The animal protocols in this experiment were approved by the Acibadem Mehmet Ali Aydinlar University Local Ethics Committee (No. HDK-2017/16, 23 April 2017).

##### Full-Thickness Excision Wound Model

Wistar Albino male rats were anesthetized with 3% isoflurane. The back of the rats was shaved and then sterilized with povidone iodine and 70% ethanol. Subsequently, a 2 × 2 cm full-thickness dorsal midline skin incision wound was made through the skin [31]. A total of 5 × 10^5^ CFU of *S. aureus* (ATCC 29213) in 12.5 µL of PBS was inoculated into the wound with a micropipette. Rats were randomly divided into four groups consisting of seven rats per group; D-TN6 (MICX20 and MICX40), ampicillin (MICX40), or SF was applied topically twice a day 24 h after *S. aureus* inoculation for 10 days. D-TN6 and ampicillin were dissolved in 100 µL of SF. The animals were housed in individual cages, and wounds were left uncovered throughout the experiments.

MPO activity was assessed as an indicator of neutrophil infiltration and inflammation. Malondialdehyde (MDA) levels, an index of lipid peroxidation, were measured using the spectrophotometric method described by Sánchez-Campillo et al. [32]. GSH levels were measured to determine the antioxidant capacity.

To determine the overall oxidative balance, the total antioxidant status (TAS) [33] and total oxidant status (TOS) [34] were measured using kits. The oxidative stress index (OSI) was calculated as TOS (μmol H_2_O_2_ equivalent/L) divided by TAS (μmol Trolox equivalent L^−1^), as described by Yumru et al. [35].

Serum levels of TNF-α, IL-1β, and IL-6 were quantified using an enzyme-linked immunosorbent assay method following the manufacturer’s instructions. Alanine transaminase (ALT), aspartate transaminase (AST), total bilirubin (TB), and C-reactive protein (CRP) levels were measured in serum samples using an autoanalyzer (Biosystems BA200 Module) and commercial assay kits.

##### Preparation of the Bacteria

Two McFarland *S. aureus* (ATCC 29213) bacteria were used for suture contamination. The 1.5 cm silk sutures (3-0) were incubated in brain heart infusion (BHI) broth at 37 °C, 150 rpm for 10 min. At the end of the incubation, the sutures were dried on sterile filter paper for 10 min. After drying, one of the sutures was placed in BHI broth, and the bacteria were measured. The number of bacteria obtained from the suture was 7 × 10^5^ CFU/mL.

##### Surgical Site Infection Model

The rats were randomly divided into four groups consisting of seven rats per group and anesthetized with isoflurane. The back of the rat was shaved, sterilized with 70% ethanol, and two 1.5 cm full-thickness wounds were incised centrally on the dorsal midline of the neck [36]. A 1.5 cm *S. aureus* (ATCC 29213)-infected 3-0 silk suture was placed into the wound incision. A staple was attached to the middle of the incision to prevent the rat from removing the infected suture. After implantation of the suture into the wound, the rats were treated topically once with 100 µL of D-TN6 (MICX40), ampicillin (MICX40), or SF. The animals were decapitated 3 h after treatment. Wound areas were dissected and placed into BHI broth. After homogenization, the bacteria on the wound were counted [36].

#### 2.2.13. Light Microscopy Tissue Processing

Skin and liver tissue specimens were fixed with 10% neutral buffered formalin solution for routine light microscopic evaluation [37]. Paraffin-embedded sections of the skin samples were scored to evaluate the degree of skin damage based on epithelial degeneration, dermal edema, hair follicle degeneration, subcutaneous degeneration, and vasocongestion (0: none; 1: low; 2: mild; 3: high), with 15 as the maximum total score [38]. Liver tissue samples were scored to evaluate the degree of liver damage based on vasocongestion, inflammatory cell infiltration, sinusoidal dilatation, fibrosis, hepatocyte degeneration, and edema (0: none; 1: low; 2: mild; 3: high), with 18 as the maximum total score [38].

#### 2.2.14. Statistical Analysis

Data were analyzed with one-way ANOVA and Tukey’s multiple comparison tests, with *p* < 0.05 considered significant. Statistical analysis was performed using GraphPad Prism 8.0 (GraphPad Software, San Diego, CA, USA).

## 3. Results

### 3.1. Molecular Dynamics Simulation

MD simulations were performed to characterize the interactions of the designed peptides with the *E. coli* membrane model and select the most active peptides for in vitro studies. As the behavioral dynamics of each molecule vary over time, the simulations were carried out for each molecule over various time periods.

**Figure 1:**
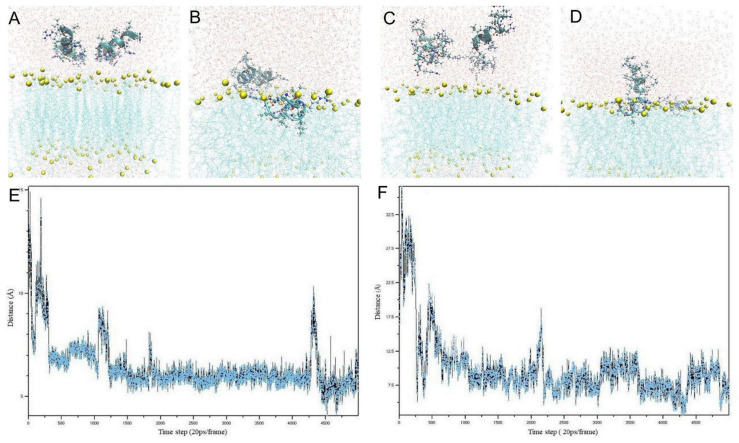
Positioning of the peptide molecules on the bacterial membrane. Side view of the D-TN6 peptide (**A**) above the membrane and (**B**) after 100 ns simulation. Side view of the D-TN6 peptide (**C**) above the membrane and (**D**) after 100 ns simulation. (**E**) Distances of the D-TN6 peptide from the membrane during the 100 ns simulation. (**F**) Distances of the TN6 peptide from the membrane during the 100 ns simulation. Water molecules are shown in red–white, phosphate molecules in yellow, membrane lipids in light blue, and the peptide backbone in turquoise. In the peptide side chains, nitrogen atoms are shown in blue, oxygen is shown in red, and hydrogen is shown in white.

During short simulations of 100 ns, the TN6 and D-TN6 peptide molecules tended to move toward and penetrate the membrane (Figure 1A–D). In these simulations, the distances of the peptide molecules from the membrane were monitored for each time step (Figure 1E,F).

### 3.2. Antimicrobial Activity of the Peptides

All the peptide sequences and MIC results are listed in Table 1. The TN3 peptide was synthesized with two different carboxy termini: one ending with an amide group and the other with carboxylic acid. The amide-end-form TN3 exhibited twofold and eightfold higher activities against *S. aureus* and *P. aeruginosa*, respectively. All other peptides were synthesized in the amide form.

Among the peptides containing only Arg and Leu, the most effective AMPs on *S. aureus* had Arg at both ends (TN1, D-TN1, TN6, D-TN6, RTN6). TN1 and TN6 had higher activities against *P. aeruginosa* when synthesized in the D-form. D/L-TN6 and TN6(2) also had Arg at both ends; however, these peptides did not follow the same trend, which may be due to differences in their alpha-helical structures.

The TN6 peptide was designed such that the Leu stretch in the middle was extended and an Arg was added to the C-terminus of TN3-amide. This peptide exhibited a fourfold higher activity against *S. aureus, E. coli*, and *C. albicans*. The D-form of this peptide was even more active against all the bacterial strains studied. A representative UPLC chromatogram and MS-MS data are given for DTN6 in Figure S1A,B. All peptides that were composed of only Arg and Leu amino acids except RTN6 had the highest activity against *C. albicans* in both the D- and L-forms.

When all the Leu amino acids in TN6 were replaced either with Ile (TN6I1) or Ala (TN6A5), the new peptides exhibited decreased activity against all other organisms studied. When all the Leu amino acids were replaced with Val in the TN3-amide peptide (TN3V9), the activity of the peptide decreased against all organisms. Even when only the Leu following Arg was replaced with valines in the TN3-amide peptide, the activity against *E. coli, P. aeruginosa*, and *C. albicans* decreased.

In addition, the activity of D-TN6 against MRSA, vancomycin-resistant *E. faecium* (VRE), *C. krusei*, *K. pneumoniae*, *A. baumannii*, *P. aeruginosa*, *C. albicans*, and *A. flavus* strains was evaluated (Table S1). The D-TN6 peptide was found to be significantly effective against all these multi-drug-resistant organisms. The MIC value of the D-TN6 peptide against several colistin-resistant *K. pneumoniae* strains was measured to determine if there may be cross-resistance to D-TN6, as colistin’s target is the bacterial membrane, but no resistance against D-TN6 was found in these strains (Table S2).

### 3.3. Evaluation of the Toxicity and Hemolytic Activity of AMPs

The toxicity profiles of the AMPs were characterized with MTT and hemolytic activity assays. HaCaT and 3t3 were exposed to peptides for 24 h at concentrations ranging from 1 µg/mL to 32 µg/mL (Figure S2A,B).

**Figure 2:**
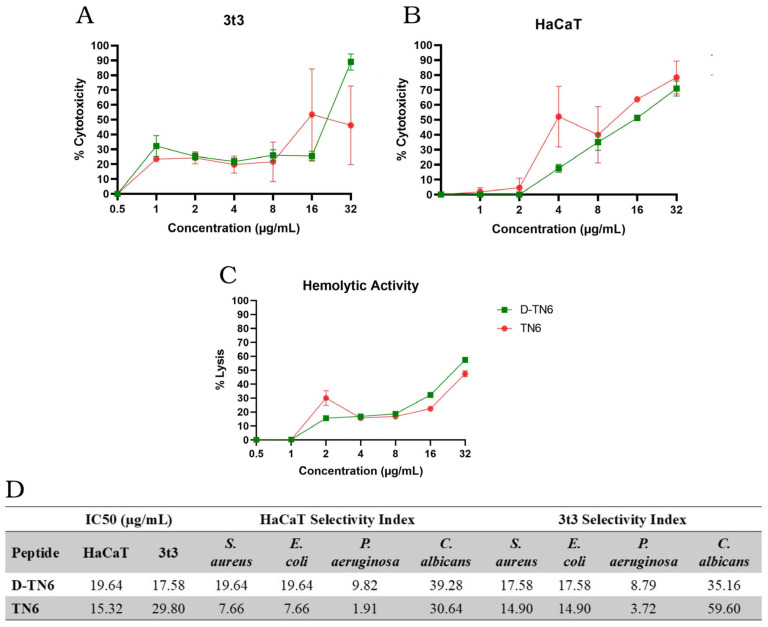
Evaluation of D-TN6 and TN6 peptides at different peptide concentrations. Cytotoxicity results against the (**A**) 3t3 cell line and (**B**) HaCaT cell line; (**C**) hemolytic activity of D-TN6 and TN6 peptides at different peptide concentrations against human erythrocytes. (**D**) Table of IC50 and selectivity indexof D-TN6 and TN6. Selectivity index is the IC50/MIC value of the related organism.

The IC50 values of both TN6 and D-TN6 determined using HaCaT and 3t3 (Figure 2A,B) were all higher than the MIC values (Table 1) of these peptides against all organisms studied. These values are given in the form of the selectivity index in Figure 2D.

Among all the peptides, D-TN6 had the lowest MIC value, which was 2 µg/mL against the *P. aeruginosa*. The D-TN6 peptide may be considered safe and non-toxic at concentrations lower than eight times the MIC value (16 µg/mL) against *P. aeruginosa* (Table 1 and Figure 2D). The selectivity index value is an important design parameter in the drug development process for therapeutic applications, and greater values are preferred for candidate drugs for further development. However, there is no universal threshold for the selectivity index because it would depend on many factors such as the efficacy of the candidate molecule or how well it would fill the gap between the current standards and unmet needs [39]. D-TN6 revealed selectivity index values from 17 to as high as 39 for both the HaCaT and 3t3 cell lines. These values are in line with the current literature [40,41,42]. For all other organisms, D-TN6 revealed selectivity index values from 17 to as high as 39 for both the HaCaT and 3t3 cell lines.

The percentage lysis values of red blood cells by the D-TN6 and TN6 peptides in the concentration range of 1 µg/mL and 32 µg/mL are shown in Figure 2C. The hemolytic activity of the D-TN1, D-TN3, and D-TN6 peptides that killed 50% of the red blood cells (HC50) was at least four times greater than their MIC values against all the organisms studied (Figure S2C and Table S3).

### 3.4. Protease Resistance Assay

**Figure 3:**
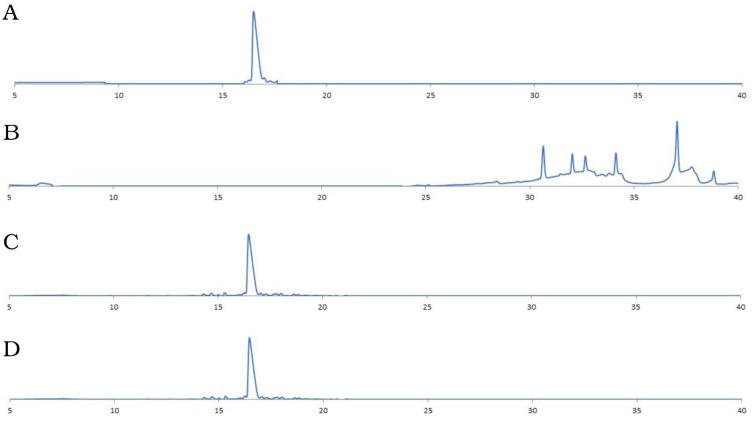
Representative HPLC chromatograms of the protease assay. TN6 peptide (**A**) before and (**B**) after protease treatment. D-TN6 peptide (**C**) before and (**D**) after protease treatment.

The TN6 peptides synthesized in the D- and L-forms were used in protease resistance assays. The analytical HPLC analysis results of the peptides after being treated with proteinase K, comparing the D- and L-forms, are given in Figure 3.

The retention time of the L-form TN6 peptide not incubated with proteinase K was 16.28 min under the given conditions (Figure 3A). The peptide disappeared after 16.28 min after incubation with proteinase K and appeared as a different species at different retention times (Figure 3B). The retention time of the D- TN6 peptide did not change before or after proteinase K incubation, suggesting that it became highly protease-resistant (16.8 min at Figure 3C,D).

### 3.5. Resistance Development Studies

In the resistance development studies with *S. aureus* ATCC 29213, the initial MIC value of gentamicin and D-TN6 was found to be 1 µg/mL. With continuous serial passaging, the MIC value of gentamicin increased to 8 µg/mL in the sixth passage. By contrast, the MIC value of the D-TN6 peptide did not change in the 11th passage.

### 3.6. Electrochemical Measurements

**Figure 4:**
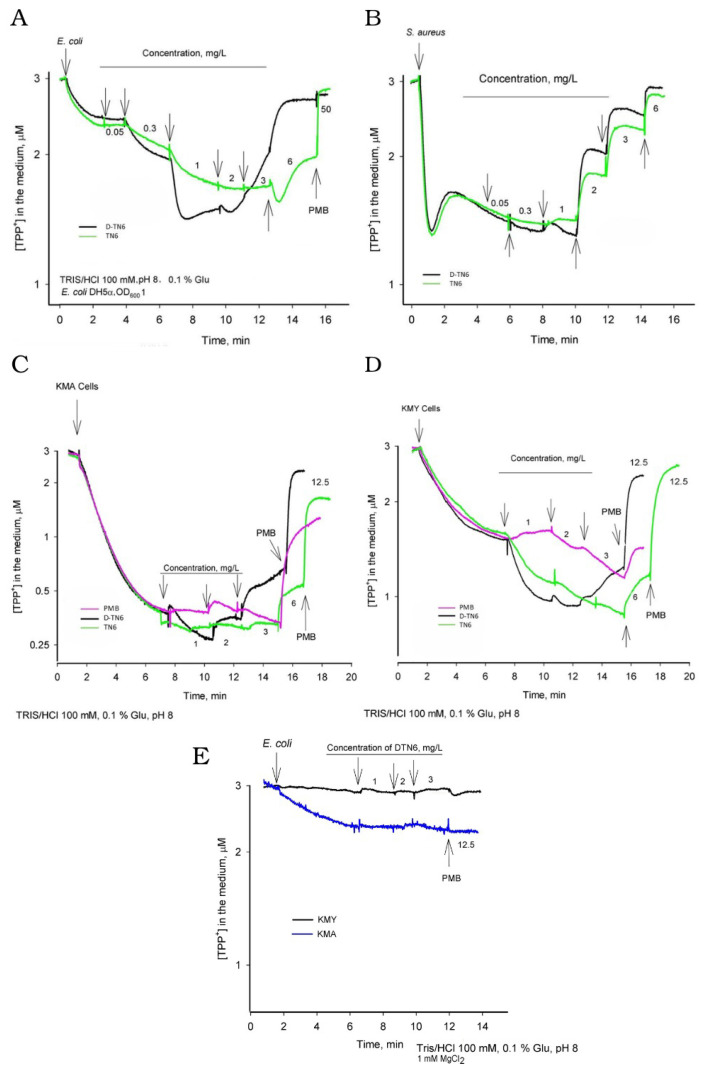
Depolarization activities of D-TN6 and TN6 peptides on cells. (**A**) *E. coli* DH5*α*; (**B**) *S. aureus* ATCC 29213; (**C**) *E. coli* KMA1; (**D**) *E. coli* KMY1 wt; (**E**) *E. coli* KMA1 and *E. coli* KMY1.

A representative set of curves from three independent experiments is presented (Figure 4). The depolarization activities of the peptides were c on *E. coli* DH5*α* cells. As shown in Figure 4A, the medium concentration of tetraphenylphosphonium (TPP+) decreased until the concentration of the peptides increased to 0.3 µg/mL. At higher concentrations of the peptides, the TPP+ concentration of the medium started to increase. This increase was at 2 µg/mL for D-TN6 and 6 µg/mL for TN6. In the experiments with *S. aureus* cells, both peptides induced depolarization of the plasma membrane (PM) at concentrations higher than 1 µg/mL (Figure 4B). The effects of the peptides on PM were also investigated in *E. coli* KMA1 cells without the main efflux pump AcrAB-TolC (KMY1 wt) (Figure 4C,D). The effects of the peptides were rather similar. The PM depolarization activity of D-TN6 was also investigated on KMA and KMY cells by supplementing the medium with MgCl_2_. This resulted in a significantly reduced activity of the D-TN6 peptide (Figure 4E).

### 3.7. Morphological Evaluation of E. coli

**Figure 5:**
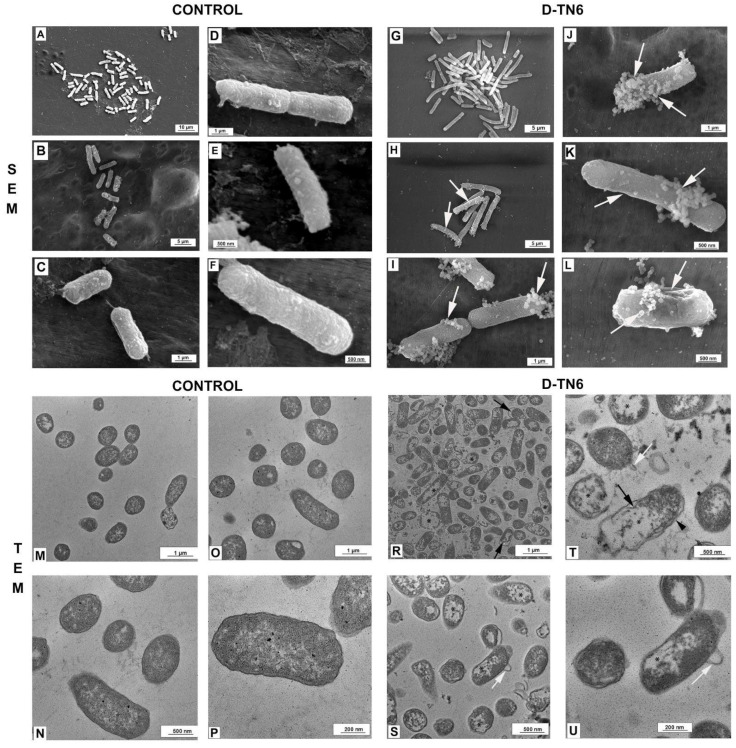
Representative SEM and TEM micrographs of the experimental groups. SEM micrographs of *E. coli* at different magnifications in the control and D-TN6 groups. Craters and burst cells (arrow) were observed in the D-TN6 group. Scales showing (**A**) 10 µm, (**B**,**G**,**H**) 5 µm, (**C**,**D**,**I**,**J**) 1 µm, and (**E**,**F**,**K**,**L**) 500 nm. TEM micrographs of *E. coli* at different magnifications in the control and D-TN6 groups. Numerous bubble-like protrusions from the cell surface (arrow), damaged cells (asterisk), and degenerated cell walls (arrowhead) were observed in the D-TN6 group. Scale bar: (**M**,**O**,**R**) 1 µm, (**N**,**S**,**T**) 500 nm, and (**P**,**U**) 200 nm.

Normal morphology was observed in the SEM micrographs of the bacterial samples in the control group (Figure 5). Long, smooth, and robust bacterial structures were observed in this group. In the control group samples, bubbles and small pits were also observed on the surface of the cells. However, the average length of the bacteria and their normal morphology remained unchanged. Small pits and blistering were observed in the samples of *E. coli* bacteria treated with D-TN6 (Figure 5).

### 3.8. Ultrastructural Evaluation of E. coli

Normal morphology was observed in the TEM microscopic images of the bacterial samples from the control group. The inner and outer membranes retained their integrity. Normal morphology was observed in the PM and cell wall. Distorted morphology was observed in the samples of *E. coli* treated with D-TN6 (Figure 5). In some bacteria, thickening of the cell membrane structure and degenerated morphology with a discontinuous appearance of the membranes and balloon-like filamentous structures extending from the cytosol to the periplasmic region were observed (Figure 5).

### 3.9. Animal Experiments

**Figure 6:**
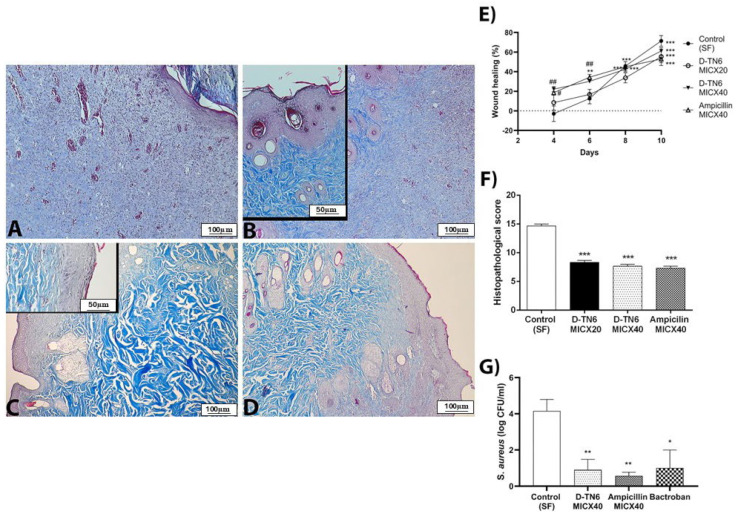
Representative photomicrographs of the skin tissue in the experimental groups. Epithelial degeneration and dermal edema were observed in the control group (**A**). Regular organization of the epidermal and dermal layers compared to the control groups with regional hair follicular disruption was observed in the (**B**) D-TN6 MICX20, (**C**) D-TN6 MICX40, and (**D**) ampicillin MICX40 groups. Masson’s trichrome staining. Scale bars are 100 µm for A, B, C, and D and 50 µm for inner images in B and C. (**E**) Wound healing (%) in rats in the D-TN6 (MICX20 or MICX40), ampicillin (MICX40), or SF-treated control groups on days 4, 6, 8, and 10 in the full-thickness excision wound model. Results are presented as means ± SEM; **: *p* < 0.01; ***: *p* < 0.001 vs. day 2. # *p* < 0.05; ##: *p* < 0.01 vs. the control group. (**F**) Skin tissue histopathological score graph. Results are presented as means ± SEM; ***: *p* < 0.001 versus control. (**G**) Infection in a rat infection model. Viable counts of bacteria in the D-TN6 (MICX40), ampicillin (MICX40), or SF-treated wound groups at 3 h post-operation. MICX20 refers to the 20-fold MIC concentration; MICX40 refers to the 40-fold MIC concentration against *S. aureus*. Results are presented as means ± SEM; *: *p* < 0.05; **: *p* < 0.01 versus the control.

The wounds treated with D-TN6 MICX40 and ampicillin MICX40 consistently exhibited smaller sizes compared with the wounds treated with SF (Figure 6E). By day 4, wound healing was significantly accelerated in the D-TN6 MICX40 and ampicillin groups in comparison to the control group (*p* < 0.01). These results indicate that D-TN6 MICX40 and ampicillin MICX40 accelerated skin wound closure and healing in rats. On the other hand, wound healing continued to accelerate on days 8 and 10 in the D-TN6 and ampicillin groups when compared to the second day (*p* < 0.001). However, there was no significant difference compared to the control group on these days.

GSH depletion in the wound tissue of the control group was prevented by D-TN6 MICX40 (*p* < 0.0001 versus control group, *p* < 0.001 vs. ampicillin MICX40 group, and *p* < 0.01 vs. D-TN6 MICX20). On the other hand, there was no difference between the groups in terms of MPO, TAS, TOS, OSI level or SOD activity, or MDA levels in the wound tissue. Treatment with D-TN6 MICX20 and MICX40 also prevented liver GSH depletion compared with the control group (*p* < 0.01). Additionally, D-TN6 MICX40 was more effective than ampicillin MICX40 (*p* < 0.01). No significant difference was found in MPO, MDA, and SOD between the groups (Table S4).

IL-1β levels were slightly reduced in the D-TN6 MICX40 group with respect to the control group in serum (*p* < 0.01). There was no significant difference in the cytokine levels between the liver and wound tissue (Table S5). D-TN6 (MICX20 and MICX40) and ampicillin MICX40 did not exhibit any indicators of toxicity, as shown by the ALT, AST, TB, and CRP levels in the serum samples (Table S6). Bacterial counts were significantly reduced in both the D-TN6 (*p* < 0.01) and ampicillin MICX40 groups (*p* < 0.01) compared with the control group (Figure 6G).

### 3.10. Histopathological Analysis

Collagen fiber disorganization, neutrophil infiltration, and hair follicle damage were observed in the dermis of the control group. In the D-TN6 MICX20 group, disorganization of the fibers was observed in the dermis. In the D-TN6 MICX40 group, epithelial degeneration was less than that in the ampicillin MICX40 group, but local disruption of fiber organization and degeneration of hair follicles were observed in the dermis (Figure 6A–D). When comparing the experimental groups with the control group, a statistically significant decrease in the histopathological score was observed (Figure 6F).

Fibrosis-like damage to hepatocytes and the periportal area was not observed in the liver tissue sections in any experimental group. Sinusoidal dilatation was observed in some areas, and regionally, more hepatocyte damage was observed in the control group when compared to the other experimental groups. Hepatocyte damage was similar in the D-TN6 MICX20, D-TN6 MICX40, and ampicillin MICX40 groups (Figure S3). The histopathological score of the control group was significantly higher compared with the other experimental groups (Figure S4).

## 4. Discussion

The AMPs developed in this study have an alpha-helical structure and contain successive hydrophobic and positively charged amino acids [43]. The TN1, TN3, and TN6 sequences, which are rich in arginine and leucine, have high activity against microorganisms and low toxicity to cells of human origin. However, they are sensitive to proteases [44]. In this study, we developed protease-resistant forms of these three peptides and modified their structure to increase their activity and decrease their toxicity. The interaction between a protein receptor and its ligand is known to be stereospecific. Thus, proteases cannot bind to D-amino acids, the enantiomer of the ligand. We have also shown previously that when conjugated to soluble polymers, these peptides can also be made protease-resistant, more soluble in water, and active against several pathogens [45].

MD simulations enabled predictions of the antimicrobial effects of the designed peptides and therefore the selection of the most probable candidate molecule while performing the experimental studies. Furthermore, it was possible to computationally analyze how the prospective drug molecule would interact with the microbial membrane. We performed MD simulations for all the peptides studied here and found a good correlation between the simulations and in vitro studies. If the peptides studied agglomerated with each other and did not tend to move toward membrane, they would show no or poor antimicrobial activity.However, when they exerted antimicrobial activity, the positively charged Arg likely interacted with the anionic phosphate groups of the phospholipid membrane, while the hydrophobic Leu helped embed the peptide in the membrane. Both the L- and D-forms of the TN6 peptide were observed to enter the membrane during the simulation period (Figure 1). Overall, it was determined that the D-TN6 peptide tended to approach and enter the membrane.

Many properties determine the antimicrobial activity of peptides, including secondary structure, net charge, peptide length, and hydrophobicity. Net charge is one of the most important contributors to antimicrobial activity. The TN3 and TN3V9 peptides have the same length and net charge of +4 at pH 7, but differences in antimicrobial activity have been shown in peptides with the same charge and length [46]. The TN3 peptide had an alpha-helical structure, whereas the alpha-helical structure was disrupted when Leu and Val were replaced. TN3 was also more hydrophobic than TN3V9. Therefore, there is no universal standard for the antimicrobial activity of peptides.

The molecule with the highest in vitro activity among the synthesized peptides was D-TN6. When TN6 was synthesized with D-amino acids, its activity increased at least twice against the standard ATCC strains used in this study. There is a slight difference in the alpha-helical structure of the L- and D-form peptides, which may account for the small changes in their activity. The MIC was 1 μg/mL against the bacterial strains *S. aureus* and *E. coli*. D-TN6 had a higher MIC against the *P. aeruginosa* strains, at 2 μg/mL. As a different sequence using the TN6 template with the D and L-form amino acids together, the arginine amino acids were retained in L-form, while the leucine amino acids were used in L- and D-forms, respectively. However, the activity of the peptide decreased when compared with both TN6 and D-TN6. According to the MIC results, D-TN6 was selected as the target molecule within the scope of the study. When examining cytotoxicity, the D-TN6 peptide was found to exceed the 50% toxicity limit. The concentration exceeding the IC50 was determined to be 17.58 μg/mL against the 3t3 cell lines and 19.64 μg/mL against the human HaCaT skin cell lines. However, despite low concentrations of cytotoxicity, the D-TN6 peptide was found to be toxic at concentrations at least 16 times the MIC value (Figure 2D). The hemolytic effect of D-TN6 was determined by the dose-dependent assay on human erythrocytes. In red blood cells, the toxicity of the D-TN6 peptide remained below the HC50 value at a concentration of 16 μg/mL (Figure 2C).

When TN6 and D-TN6 were treated with proteinase K, the retention time of the D-peptides remained the same (Figure 2B,D). Proteinase K is an enzyme that can digest a range of proteins, including those that are resistant to other proteases. It achieves this by hydrolyzing the peptide bonds on the carboxyl side of the amino acids that contain aromatic, aliphatic, or hydrophobic amino acid residues [28]. The proteinase-K-treated TN6 peptide did not arrive at the retention time of the intact molecules but produced small peaks at different retention times. This proved that the L-peptide was cleaved by the protease treatment while the D-peptide remained intact. While D-TN6 was resistant to protease, TN6 was fragmented into different-sized molecules in the HPLC analysis. In the proteolytic resistance studies, the D-TN6 peptide was resistant to proteinase K after 10 min, 30 min, and overnight incubation (Figure 3C,D), whereas TN6 was fragmented into different-sized molecules, as shown by HPLC (Figure 3A,B). When the MIC assay for the proteinase-K-treated D-TN6 peptide was repeated, the peptide retained its activity at the same concentration. It is possible that the increased protease resistance of D-TN6 has wider implications for its stability under physiological conditions. These may include the following: (i) the peptide can remaining intact for a longer duration in the presence of proteolytic enzymes found in biological fluids to enhance its bioavailability and therapeutic efficacy over a prolonged period, or (ii) it may require less frequent administration compared to more susceptible peptides, improving patient compliance in therapeutic settings. However, while stability is beneficial, there is a potential risk of off-target effects or toxicity if the peptide remains active for too long.

The bactericidal effect is often explained by the contact between the peptide and the bacterial cell membrane, leading to the formation of holes that can result in cell lysis [47]. Experimental studies have also attributed the effect of AMPs on bacteria to the inhibition of bacterial cell wall biosynthesis, alterations to membrane permeability, and impairment of nucleic acids and proteins, leading to cell lysis and death [48,49]. To investigate the effects of the AMP D-TN6 on *E. coli*, we performed an ultrastructural analysis. The morphology of *E. coli* cells changed after treatment with D-TN6; small pits and blistering were observed in the D-TN6-treated samples of *E. coli*. Thickening of the cell membrane and degenerated morphology with a discontinuous appearance of membranes and balloon-like filamentous structures were also observed in the D-TN6-treated *E. coli* bacteria. In particular, the filamentous structures may play a role in the bactericidal processing of the peptide. The disruption of the bacterial cell membrane can lead to morphological alterations, such as balloon-like structures, which may be related to cellular damage or stress responses induced by the AMP [50]. Similar morphological changes have been reported with other AMPs such as gramicidin S and PGLa [51]. These alterations are associated with the ability of peptides to disrupt the bacterial cell envelope and compromise cell integrity [51].

These morphological changes were further investigated with electrochemical measurements. The bacterial cells are negative inside because of the transmembrane difference in the electrical potential on the PM. However, a lipopolysaccharide-built outer layer of the OM and the efflux pumps prevent the entry of TPP+ cations into the cells. As shown in Figure 4A, the cells started accumulating TPP+, and the concentration of this indicator in the medium began to decrease when the medium concentration of the peptides increased to 0.3 µg/mL. The accumulation of TPP+ by the cells indicated that the peptides started to permeabilize the outer membrane at this concentration. At higher concentrations, the peptides induced leakage of accumulated TPP+ out of the cells back into the medium, indicating the plasma-membrane-depolarizing activity of the peptides. D-TN6 started to show depolarizing activity at 2 µg/mL and TN6 at 6 µg/mL.

Gram-positive *S. aureus* cells have only one PM. TPP+ flux measurements with *S. aureus* indicated that both the TN6 and D-TN6 peptides induced depolarization of the PM at 1 µg/mL. These results indicate that the OM of Gram-negative cells form a stronger barrier to TN6 than D-TN6 (Figure 4).

The activity of D-TN6 was also investigated on *E. coli* KMA1 and KMY1 cells incubated in 100 mM sodium phosphate (pH 8.0) or in 100 mM Tris/HCl (pH 8.0) supplemented with 1 mM Mg^2+^. In both cases, the activity of D-TN6 was considerably lower. This indicates that the increased ionic strength of the medium inhibits the action of the peptide, likely preventing the peptide from binding to the cell surface (Figure 4E).

Inflammation plays a pivotal role in the initial phases of wound healing; moderate levels aid in the healing process [44,52]. In the *S. aureus*-induced model, we investigated the effects of D-TN6 on inflammation during wound tissue healing. AMPs enhance wound healing in the skin by modulating cytokine production, cell migration, proliferation, and in rare cases, angiogenesis [53]. The wound area of each group decreased over time. However, wounds in the D-TN6-treated rats closed significantly faster on the fourth and sixth days. On the eighth day, the difference in wound sizes among the groups became negligible. The beneficial effects of AMPs on wound healing extend not only to the skin but also to other related tissues, such as the stratified non-keratinized corneal epithelium and the glands of the eyelids, whose development is similar to that of skin appendages such as hair follicles and sebaceous glands [54]. In our study, disorganized fibers in the dermis were observed in the D-TN6 MICX20 group. In addition, epithelial degeneration was less than that in the ampicillin MICX40 group, but local disorganization of fibers and degeneration of hair follicles in the dermis were observed in the D-TN6 MICX40 group.

Here, administration of high-dose D-TN6 elevated GSH levels in the wound tissue and liver compared with the control group. Histological evaluation of liver parameters tended to decrease in the MICX40 group compared with the control group. These observations show that high doses of D-TN6 enhance antioxidant activity, both systemically and locally. Upon measuring cytokine levels in the serum, it was found that the administration of a high dose of D-TN6 led to a decrease in IL1-β levels. The histological and biochemical parameters showed the low toxic effects of the DTN-6 peptide on the liver.

Wound-healing peptides regulate anti-inflammatory properties that help the body control inflammation, resist bacterial infections, and accelerate the transition through the inflammatory phase. This, in turn, enables the progression of wound healing into the subsequent stages, potentially enhancing the recovery of chronic and infected wounds [55]. In the presence of the peptide, reduced oxidative stress via increased levels of GSH, along with decreased IL1-β levels, may have also accelerated wound closure and healing.

In our study, the liver and skin tissue exhibited nearly normal morphology. The liver tissue hepatocytes and stratified epithelium did not exhibit any toxic alterations, suggesting a favorable safety profile for D-TN6 and bodes promise for advancement to clinical trials. Future in vivo research should include dose-escalation studies to determine the maximum tolerated dose in rats and humans. The findings here can guide future clinical studies on the efficacy and safety of D-TN6.

In addition to the full-thickness excision model, a surgical site infection model was performed in rats to explore how D-TN6 affects the bacterial count. In this model, a silk suture contaminated with *S. aureus* was inoculated into an incision wound on the back of the rats, and the course of infection was assessed by counting viable bacteria in the tissue homogenate. Topical application of D-TN6 significantly reduced bacterial counts in the wound tissue, consistent with our in vitro findings. Similar results were reported in a previous study on a surgical site infection model, in which application of a novel short synthetic AMP decreased bacterial counts in the wound tissue at various post-surgical time points [44].

The protease-resistant AMPs produced in this study are promising drug candidates due to their high antimicrobial activity and low mammalian cell toxicity. The activity of AMPs was tested with bacteria commonly used as a standard in antimicrobial activity experiments. These bacteria are known to be capable of developing resistance to the antibiotics used for the treatment. Although D-TN6 exhibited a low-risk profile in the skin model here, the following should be considered for assessing its risk profile in clinical trials: (i) The drug may be absorbed, distributed, metabolized, and excreted differently in in vivo compared to the models used in this study. This may lead to unexpected side effects or reduced efficacy. (ii) The ability of the AMP to penetrate host tissues and reach the site of infection may differ from the study conditions, thereby affecting its effectiveness. (iii) The immune response to the antibiotic, though not observed in a rat skin model, including hypersensitivity reactions, can introduce risks that are not apparent in animal studies.

The efficacy of the D-TN6 peptide against “ESKAPE pathogens”, multi-drug-resistant *A. flavus* (AF), and *C. albicans* (CA) strains was investigated. The World Health Organization first published their “Bacterial Priority Pathogens List” in 2017. Since that initial publication, carbapenem-resistant *A. baumannii* has been classified into the critical group and vancomycin-resistant *E. faecium* and methicillin-resistant *S. aureus* have been classified into the high-priority group as resistant isolates [56]. The D-TN6 peptide has been tested against these bacteria that threaten human health and has been shown to have an inhibitory effect. In MIC studies, the D-TN6 peptide was found to be effective from 0.25–4 μg/mL against these seven species that are resistant to current antibiotics (Table S1).

A comparative experiment with gentamicin was also performed to see if bacteria could develop resistance to the D-TN6 peptide. With continuous serial passaging, it was observed that the bacteria developed resistance to gentamicin at the sixth generation, and the MIC value increased to 8 μg/mL. In simultaneous experiments with D-TN6, the bacteria failed to develop resistance at the 11th generation, and the MIC value remained at 1 μg/mL. These results showed that even if the bacteria could develop resistance to D-TN6, such development would be much slower than the development of resistance to gentamicin, which is in clinical use. The SEM and TEM microscopy results showed that the membranes of the D-TN6-treated bacteria started to deteriorate. These deteriorations disrupted the integrity of the bacterial membranes, causing them to disintegrate. With just a single mutation, bacteria can develop resistance to antibiotics that target a specific protein or ribosomes. However, it is very difficult for bacteria, though not impossible, to develop resistance to antibiotics that target bacterial membranes because membrane lipids cannot be changed with a single mutation. Resistance to colistin, which targets bacterial membranes, mostly develops by alteration of the lipopolysaccharides and a reduction in its negative charge [57]. Because D-TN6 contains positively charged amino acids that seem crucial for its activity, it may be thought that the same mechanism may reduce its activity. However, we observed no resistance to D-TN6 in several colistin-resistant *K. pneumoniae* strains in this study. This indicates that there is no cross-resistance with colistin resistance.

Compared to AMPs developed in recent years, the D-TN6 peptide developed here has superior properties in terms of its efficacy against MDR bacteria. The AP19 peptide developed by Jariyarattanarach et al. is an α-helix peptide found to be effective against bacteria [58]; it was synthesized with D-amino acids to render them resistant to proteases. The D-AP19 peptide was tested with clinical *A. baumannii* strains, with the lowest MIC value being 3.91 µg/mL. It was not toxic at MIC values and MIC × 2 values. On the other hand, the D-TN6 peptide was found to be effective in the range of 0.25–2 µg/mL against clinical *A. baumannii* strains. In addition, the D-TN6 peptide was not toxic even at MIC × 17 values (the novel D-form of the hybrid peptide (D-AP19) rapidly kills *A. baumannii* while tolerating proteolytic enzymes). Li et al. used a peptide language-based deep generative framework (deep AMP) for identifying potent, broad-spectrum AMPs [59]. The T2-9 peptide showed the strongest antibacterial activity (against *E. coli*, *S. aureus*, *K. pneumoniae*, *P. aeruginosa*, and MRSA), comparable to FDA-approved antibiotics. The activity of the DTN6 peptide developed in this study was higher against all the pathogens studied with T2-9 [59]. Another advantage of the D-TN6 peptide is its efficacy against various resistant fungal strains at low concentrations (2 µg/mL) (Table S1). In neutropenic patients with sepsis, appropriate antimicrobial treatment is urgent. Generally, an empirical therapy with a wide spectrum of antibiotics is initiated; if the signs of infection do not subside, an antifungal drug is added until the specific infectious agent is determined [60]. D-TN6, which is effective against both bacteria and fungi, may replace this combination therapy and eliminate the side effects of these conventional drugs. One of the disadvantages of AMPs is their susceptibility to proteases. Their half-life in the body is very short due to rapid digestion by proteases. They cannot be used orally because the stomach and intestines contain many active proteases. However, because D-TN6 is protease-resistant, it has the potential to be used as an oral as well as a parenteral drug. Its half-life is expected to be much longer than that of the natural peptide antibiotics produced in the human body, which are composed of L-amino acids and therefore susceptible to proteases.

## 5. Conclusions

The D-TN6 peptide is effective in vivo against standard *S. aureus* ATCC 29,213 strains in the context of wound healing. These properties make D-TN6 a broad-spectrum and potent antibiotic candidate. The efficacy of the D-TN6 peptide against microorganisms that threaten human health worldwide can help combat increased antibiotic resistance.

## Figures and Tables

**Table 1:** MIC (µg/mL) values of AMPs against *S. aureus* ATCC 29213, *E. coli* ATCC 25922, *P. aeruginosa* ATCC 27853, and *C. albicans* ATCC 10231.

Peptide	Sequence	*S. a* *ureus*	*E. coli*	*P. aeruginosa*	*C. albicans*
TN1	RLLRLLLLRLLR	4	2	32	0.5
D-TN1	**RLLRLLLLRLLR**	4	8	4	1
TN3-amide	RLLRLLRLLL	8	8	4	2
TN3-carboxy	RLLRLLRLLL	16	4	32	1
D-TN3	**RLLRLLRLLL**	8	2	4	0.25
TN3V1	RVLRVLRVLL	8	16	32	4
TN3V9	RVVRVVRVVV	16	64	256	128
TN6	RLLRLLLRLLR	2	2	8	0.5
D-TN6	**RLLRLLLRLLR**	1	1	2	0.5
TN6I1	RIIRIIIRIIR	16	32	128	128
TN6I2	RILRILIRLIR	2	16	32	128
TN6A1	RALRALARALR	128	128	256	256
TN6A5	RAARAAARAAR	>1024	1024	>1024	128
RTN6	RRLLRLLLRLLR	1	2	8	4
D/L-TN6	RL**L**R**L**L**L**R**L**LR	8	8	16	8
TN6(2)	RLLRLLRLLLRLLRLLR	16	16	64	32
TN8	RLLRLLRLLLL	8	4	256	0.5

Bold red color represents D-form amino acids.

## Data Availability

All the data are included in the manuscript.

## Supplementary Materials

The following supporting information can be downloaded at: https://www.mdpi.com/article/10.3390/life15020242/s1, Figure S1. (A) UPLC-UV chromatogram and (B) MS/MS spectrum of D-TN6 peptide. Figure S2. Evaluation of designed peptides at different peptide concentrations. With Magainin cytotoxicity results (A) Human skin keratinocyte (HaCaT) cell line and (B) Mouse embryonic fibroblast (3T3) cell line; (C) Hemolytic activity of designed peptides at different peptide concentrations on human erythrocytes. Designed peptides are D-TN1, TN1, D-TN3, TN3, D-TN6 and TN6. Figure S3. Representative photomicrographs of liver tissue in experimental groups. Sinusoidal dilation (*) and hepatocyte damage (→) in liver tissue sections of the experimental groups (A–D). A: control group; B: D-TN6 MIC 20, C: D-TN6 MIC X40, D: Ampicillin MIC 40 groups Masson’s trichrome staining. Scale bar: 50 µm. Figure S4. Liver tissue histopathological score graph. Results were presented as means ± S.E.M. **: *p* < 0.01 vs. control. Table S1. Minimal Inhibitory Concentration (µg/ml) Results of D-TN6 on resistant clinical samples (methicillin-resistant *S. aureus* (MRSA), *Aspergillus flavus* (AF), *C.albicans* (CA), vancomycin-resistant *Enterococcus faecium* (VRE), *C.krusei* (CK), *Klebsiella pneumoniae* (KP), *Acinetobacter baumannii* (AB)). Table S2. Minimal Inhibitory Concentration (µg/ml) Results of D-TN6 and POLB on resistant *Klebsiella pneumoniae* (KP) strain. Table S3. Table of HC50, and safety index* of D-TN1, D-TN3, and D-TN6. Table S4. MPO, SOD, MDA, GSH, TAS, TOS, OSI values in all groups using the full-thickness excision wound model. Table S5. TNF-α, IL1-β, and IL-6 value in all groups. Table S6. Serum ALT, AST, TB and CRP levels.
