# Supplementary material for: Protease-Resistant, Broad-Spectrum Antimicrobial Peptides with High Antibacterial and Antifungal Activity
Source: Life (Basel). 2025 Feb 5;15(2):242. doi: 10.3390/life15020242 (PMC11856857; doi:10.3390/life15020242)
Supplement: Supplementary file 1 [file life-15-00242-s001.zip › life-3342194-supplementary.pdf]

Supplementary Materials

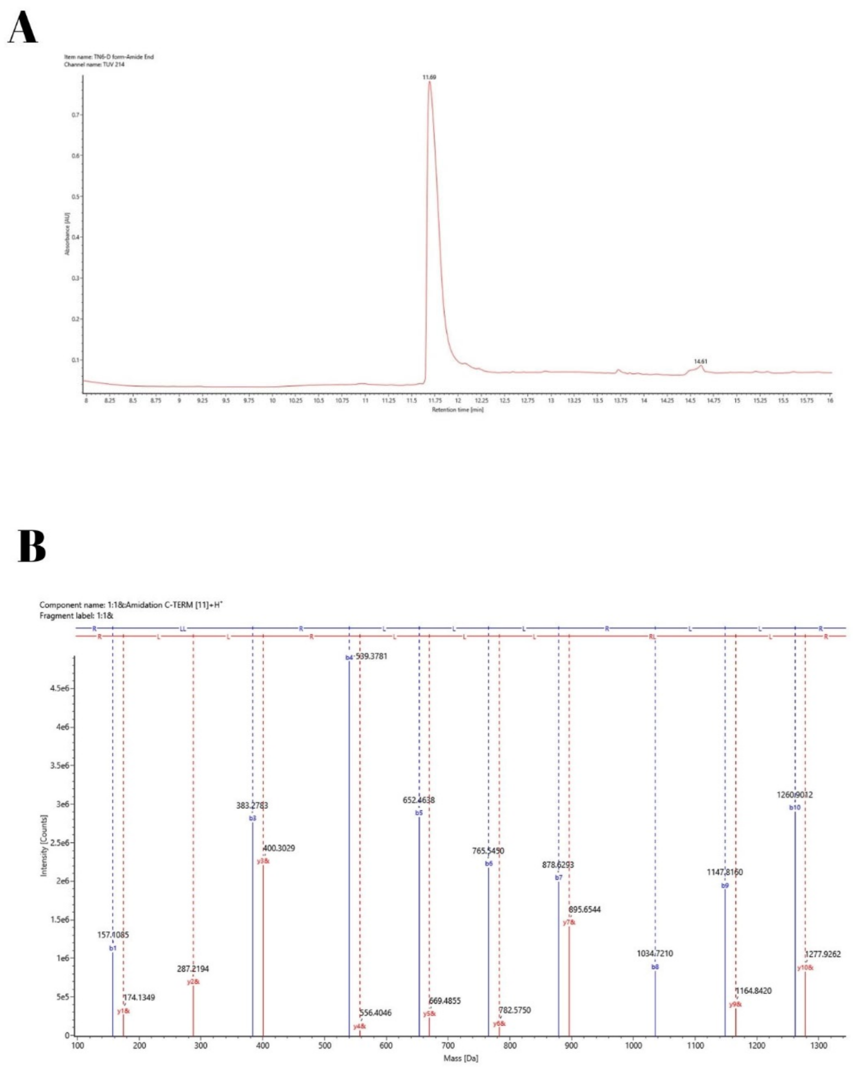

Figure S1. (A) UPLC-UV chromatogram and (B) MS/MS spectrum of D-TN6 peptide.

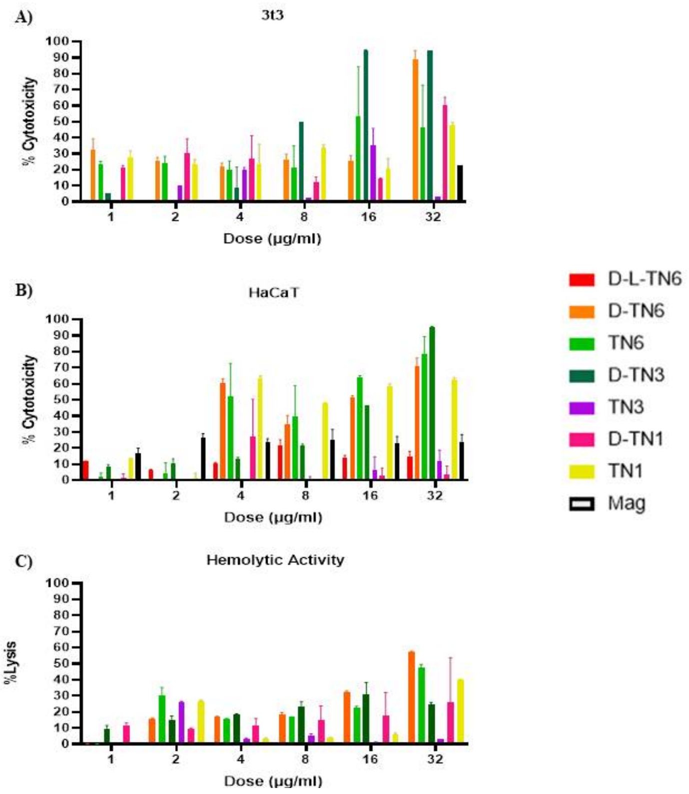

Figure S2. Evaluation of designed peptides at different peptide concentrations. With Magainin cytotoxicity results (A) Human skin keratinocyte (HaCaT) cell line and (B) Mouse embryonic fibroblast (3T3) cell line; (C) Hemolytic activity of designed peptides at different peptide concentrations on human erythrocytes. Designed peptides are D-TN1, TN1, D-TN3, TN3, D-TN6 and TN6.

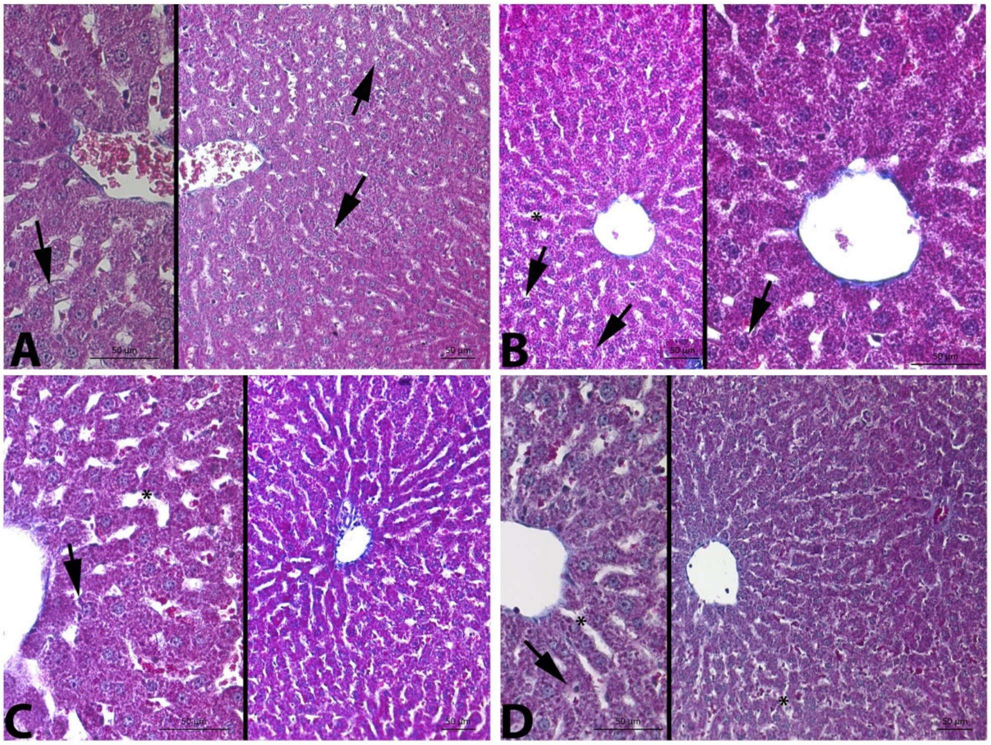

Figure S3. Representative photomicrographs of liver tissue in experimental groups.

Sinusoidal dilation (*) and hepatocyte damage (→) in liver tissue sections of the experimental groups **(A-D)**. A: control group; B: D- TN6 MIC 20, C: D-TN6 MIC X40, D: Ampicillin MIC 40 groups Masson's trichrome staining. Scale bar: 50µm

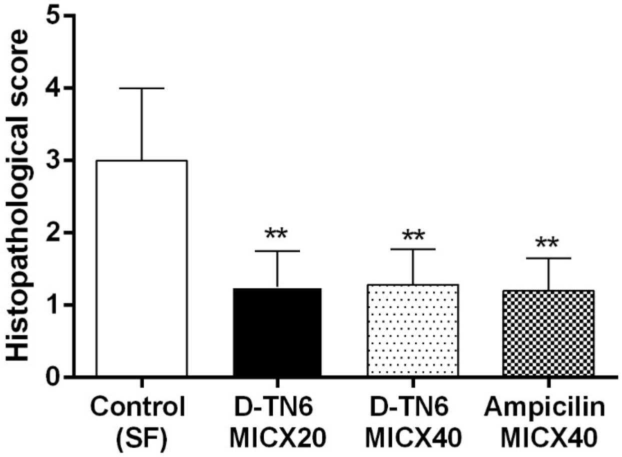

Figure S4. Liver tissue histopathological score graph. Results were presented as means ± S.E.M. **: p<0.01 vs. control.

Table S1. Minimal Inhibitory Concentration (µg/ml) Results of D-TN6 on resistant clinical samples (methicillin-resistant *S. aureus* (MRSA), *Aspergillus flavus* (AF), *C.albicans* (CA), vancomycin-resistant *Enterococcus faecium* (VRE), *C.krusei* (CK), *Klebsiella pneumoniae* (KP), *Acinetobacter baumannii* (AB)).

Bacterial Strain	D-TN6	Bacterial Strain	D-TN6
**MRSA 1**	0.5	**CK 1**	2
**MRSA 2**	1	**CK 2**	2
**MRSA 3**	1	**CK 3**	2
**AF1**	4	**KP 1**	4
**CA 1**	2	**KP 2**	2
**CA 2**	2	**KP 3**	1
**CA 3**	1	**KP 4**	1
**VRE 1**	0.25	**PA1**	1
**VRE 2**	0.25	**PA2**	1
**AB1**	0.25	**PA3**	0.5
**AB2**	2	**PA4**	1

Table S2. Minimal Inhibitory Concentration (µg/ml) Results of D-TN6 and POLB on resistant *Klebsiella pneumoniae* (KP) strain.

Bacterial Strain	Polymyxin B	D-TN6
**KP2**	2	1
**KP9**	2	0.5
**KP40**	4	2
**KP41**	4	4
**KP22**	8	0.5
**KP35**	8	8
**KP42**	8	2
**KP52**	8	2
**KP1**	16	0.5
**KP13**	16	0.5
**KP28**	16	1
**KP29**	16	2
**KP46**	16	2
**KP8**	64	1

Table S3. Table of HC50, and safety index* of D-TN1, D-TN3, and D-TN6.

		**Safety Index**			
**Peptide**	**HC50 (µg/mL)**	***S. aureus***	***E. coli***	***P. aeruginosa***	***C. albicans***
**D-TN1**	77.63	19.40	9.70	19.4	77.63
**D-TN3**	32.63	4.07	16.31	8.15	130.52
**D-TN6**	27.28	27.28	27.28	13.64	54.56

Table S4. MPO, SOD, MDA, GSH, TAS, TOS, OSI values in all groups using the full-thickness excision wound model.

	MPO	SOD	MDA	GSH	TAS	TOS	OSI
**Wound Tissue**							
Control (SF)	32.60 ± 6.87	657.8 ± 34.25	32.13 ± 6.36	10.78 ± 3.09	0.76 ± 0.08	3.50 ± 0.55	0.52 ± 0.16
D-TN6 MICX20	26.60 ± 4.27	759.2 ± 34.84	20.98 ± 6.04	13.95 ± 3.35	0.75 ± 0.07	2.52 ± 0.43	0.35 ± 0.06
D-TN6 MICX40	30.00 ± 3.24	653.7 ± 26.58	40.81 ± 4.84	32.20 ± 2.34**** &&& ###	0.99 ± 0.11	4.17 ± 0.43	0.45 ± 0.07
Ampicillin MICX40	29.00 ± 2.46	659.1 ± 55.63	28.69 ± 5.22	15.91 ± 1.54	0.87 ± 0.07	4.72 ± 0.45	0.57 ± 0.08
**Liver**							
Control (SF)	71.80 ± 16.42	498.8 ± 38.64	34.81 ± 10.14	0.83 ± 0.35	1.94 ± 0.07	17.78 ± 0.87	0.92 ± 0.07
D-TN6 MICX20	44.80 ± 4.13	575.8 ± 12.69	31.13 ± 3.80	1.94 ± 0.49**	1.95 ± 0.06	18.95 ± 1.11	0.98 ± 0.07
D-TN6 MICX40	61.86 ± 9.74	522.9 ± 35.51	25.46 ± 1.22	3.67 ± 0.76** ##	1.76 ± 0.90	19.53 ± 1.34	1.15 ± 0.13
Ampicillin MICX40	65.43 ± 5.68	499.6 ± 20.22	24.29 ± 0.70	0.84 ± 0.20	1.87 ± 0.05	18.55 ± 0.91	0.99 ± 0.07

Each group consisted of 8-9 rats. Results were presented as means ± S.E.M.

**: $p<0.01$ vs. the control group. ***: $p<0.001$ vs. the control group. ****: $p<0.0001$ vs. the control group. ###: $p<0.001$ vs the Ampicillin group. &&&: $p<0.001$ comparison of treatment groups.

Table S5. TNF-α, IL1-β, and IL-6 value in all groups.

	TNF-α	IL1-β	IL-6
**Wound Tissue**			
Control (SF)	169.0 ± 27.67	1559 ± 128.6	72.79 ± 5.64
D-TN6 MICX20	229.1 ± 17.81	2006 ± 123.4	88.75 ± 8.23
D-TN6 MICX40	220.1 ± 11.34	1772 ± 123.8	83.05 ± 7.70
Ampicillin MICX40	225.4 ± 12.19	1779 ± 45.54	77.60 ± 4.77
**Liver**			
Control (SF)	120.6 ± 12.03	824.7 ± 84.54	34.30 ± 4.52
D-TN6 MICX20	95.73 ± 4.89	560.6 ± 82.14	31.77 ± 1.79
D-TN6 MICX40	98.3 ± 10.36	670.2 ± 114.2	35.70 ± 3.89
Ampicillin MICX40	107.0 ± 8.14	705.7 ± 87.07	35.51 ± 2.82
**Serum**			
Control (SF)	219.7 ± 7.69	1132 ± 182.3	50.84 ± 5.57
D-TN6 MICX40	209.1 ± 6.53	607.2 ± 51.25**	45.66 ± 1.7
Ampicillin MICX40	212.2 ± 9.02	826.9 ± 105.5	50.02 ± 2.04

Each group consisted of 8-9 rats. Results were presented as means ± S.E.M.

**: p<0.01 vs. the control group.

Table S6. Serum ALT, AST, TB and CRP levels.

	ALT (U/L	AST (U/L)	TB (mg/dl)	CRP
Control (SF)	57.6 ± 3.1	110.4 ± 6.7	0.2	N
D-TN6 MICX20	68.4 ± 10.8	187.2± 26.4	0.2	N
D-TN6 MICX40	55.1 ± 6.3	199.7 ± 47.7	0.1	N
Ampicillin MICX40	52.4 ± 3	161.6 ± 12.8	0.1	N
Reference range	17 - 77	54 - 298	0.0 - 0.9	Negative

Each group consisted of 8-9 rats. Results were presented as means ± S.E.M.
